# From silos to synergies: harnessing the power of sexual health data sources in England to understand changing epidemiology and inform policy

**DOI:** 10.1136/sextrans-2025-056754

**Published:** 2026-06-08

**Authors:** Dana Ogaz, Anika Knuppel, Emily Trickett, Soazig Clifton, Charles Beck, Magnus Unemo, Pam Sonnenberg, Hamish Mohammed, Catherine H Mercer, Kate Soldan, Katy M Turner, Nigel Field

**Affiliations:** 1 Institute for Global Health, University College London, London, England, UK; 2 Blood Safety, Hepatitis, STIs and HIV Division, UK Health Security Agency, London, UK; 3 School of Health and Wellbeing, University of Glasgow, Glasgow, UK; 4 Health and Biomedical Surveys Team, National Centre for Social Research, London, UK; 5 Evaluation and Epidemiological Sciences Division, UK Health Security Agency, London, UK; 6 WHO Collaborating Centre for Gonorrhoea and Other STIs, Department of Laboratory Medicine, Faculty of Medicine and Health, Örebro University, Örebro, Sweden

**Keywords:** SEXUAL HEALTH, HIV, POLICY, Epidemiology

## Abstract

**Objectives:**

Following major changes to sexual health provision in England in the past decade, monitoring and evaluation of population-level interventions require re-examination to ensure quality and visibility of key communities. We make the case that sexual health data sources might be used synergistically to improve sexual health and tackle inequalities.

**Methods:**

We conducted a purposive narrative review to (1) describe national sexual health policy and provision changes in England in the past decade (2012 onwards), (2) map key sexual health data resources and (3) demonstrate how integrated data-driven approaches can be used to understand and evaluate population-level sexual health policy.

**Results:**

There have been major changes at a national and regional level in the availability, targeting and implementation of HIV and sexually transmitted infection (STI) interventions, including through high-level shifts in funding structures, expansion of online services and pathogen-focused policies. Synergistic use of the many data sources available takes advantage of their different features. For example, for HIV, integration of nationally representative behavioural surveys that include people not accessing care (eg, National Surveys of Sexual Attitudes and Lifestyles), targeted surveys (eg, Reducing inequalities in Sexual Health surveys for gay, bisexual and other men who have sex with men (GBMSM)) and national STI/HIV surveillance for people accessing care (eg, GUMCAD STI Surveillance System) can be used to inform progress towards HIV elimination in England through assessment of online and postal-based self-sampling and inequalities in HIV testing uptake in populations and key communities such as GBMSM.

**Conclusion:**

In the face of rapid change, approaches that integrate complementary data sources have the potential to harness the strengths of individual assets and mitigate limitations to form a more complete assessment of population-level interventions and should guide sexual health policy and practice in the decade to come. Effective data integration requires addressing a range of methods, mandates and challenges to strengthen contemporary research, monitoring and evaluation.

**WHAT IS ALREADY KNOWN ON THIS TOPIC**

- There have been marked changes in sexual health policy, provision and outcomes in England over the past decade, which prompted examination of data sources for monitoring, evaluation and research. Data integration opportunities exist but have not been widely exploited.

**WHAT THIS STUDY ADDS**

- This purposive, narrative review, (1) describes national sexual health policies introduced in England over the past decade (2012 onwards), (2) outlines key sexual health data resources available to understand sexual health at national level and (3) describes opportunities for data integration using exemplars to demonstrate how complementary data sources can be used to understand and evaluate population-level sexual health policy.

**HOW THIS STUDY MIGHT AFFECT RESEARCH, PRACTICE OR POLICY**

- This review summarises changes in sexual health policy and provision and describes key sexual health data sources and how they might be used synergistically to inform sexual health policy and provision in the decade to come. It highlights the opportunities and challenges of data synergy and encourages sexual health researchers and policymakers to consider data integration approaches for more robust sexual health research, monitoring and evaluation.

## Background

Sexually transmitted infections (STIs), including HIV, remain a significant public health challenge globally and within the UK. Despite gains in HIV prevention through expansion of combination prevention,^1 2^ HIV and bacterial STI diagnoses disproportionately impact key communities, with trends such as rising syphilis cases among heterosexuals,^3^ shigella outbreaks in gay, bisexual and other men who have sex with men (GBMSM),^4^ and persistent health inequalities^5 6^ highlighting gaps in current prevention strategies.

Since the 2012 Health and Social Care Act, sexual health services (SHSs) have experienced substantial funding cuts.^7^ Although the public health grant increased in 2025,^8^ this followed years of disinvestment. Between 2015–2022, spending on STI testing and treatment fell by 10% from £369.4 million to £332.5 million (absolute value)^9^. As local governments adjusted to reduced budgets, SHSs were heavily affected. Service delivery also changed markedly, a shift accelerated by the COVID-19 pandemic. Online services have become the initial point of contact for many and now account for nearly half of all sexual health consultations.^3^ Today, services are delivered digitally and in-person across sexual health, reproductive health, primary, community and secondary care. This mixed model adds complexity, reflected in diverse data sources and poses considerable challenges for evidence-based policymaking and undertaking robust evaluations of sexual health interventions.

The UK benefits from world-leading national surveillance datasets and population-based sexual health research surveys that have long informed intervention design, policy recommendations and enabled public health evaluation.^10 11^ However, these data sources are rarely analysed together, which we argue here is an important missed opportunity to enable more effective policy development and implementation and should be considered in developing a national sexual and reproductive health strategy.^12^

This narrative review sets out to, (1) describe national sexual health policy and provision changes introduced over the past decade (2012 onwards), (2) outline key sexual health data resources and (3) demonstrate how data integration and data-driven approaches can be used to understand and evaluate population-level sexual health policy. This review highlights the conceptual benefits of data synergy to encourage sexual health researchers and policymakers to explore the methods, mandates and practical challenges of implementing integration in their own contexts.

## Methods

We conducted a purposive, narrative review of sexual health data sources and policies in England, informed by consultation with STI, HIV and blood-borne virus (BBV) experts at the UK Health Security Agency (UKHSA) and stakeholders affiliated with the National Institute for Health and Care Research Health Protection Research Unit in Blood Borne and Infectious Diseases led by University College London and in partnership with UKHSA (https://bbsti.hpru.nihr.ac.uk). We also reviewed national policy documents, routine surveillance reports and sexual health guidelines. Data sources were summarised by coverage, structure, strengths and limitations.

## Shifts in sexual health policy and provision since 2012

**Figure 1:**
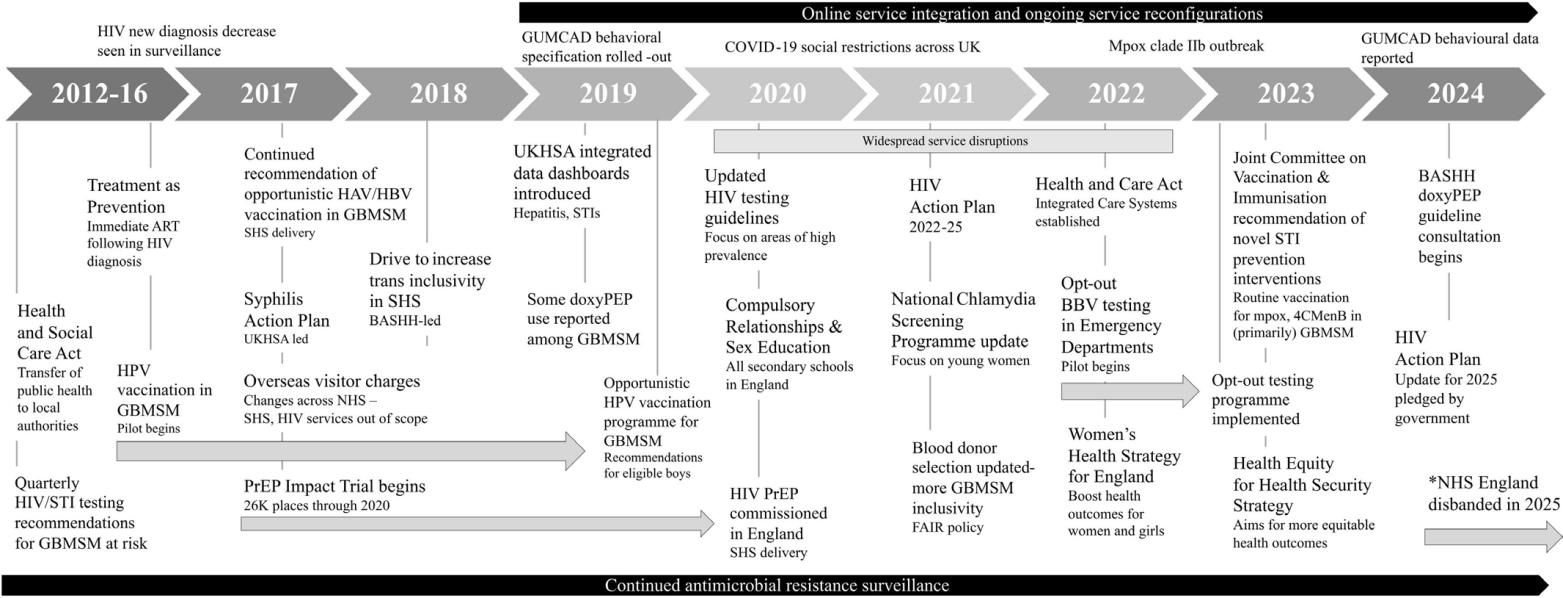
Major sexual health policy and provision changes in England (2012–2024). ART, antiretroviral therapy; BASHH, British Association for Sexual Health and HIV; BBV, blood-borne virus; doxyPEP, doxycycline postexposure prophylaxis; FAIR, For the Assessment of Individualised Risk; GBMSM, gay, bisexual and other men who have sex with men; GUMCAD, GUMCAD STI Surveillance System; HAV, hepatitis A virus; HBV, hepatitis B virus; NHS, National Health Service; PrEP, pre-exposure prophylaxis; SHS, sexual health service; STI, sexually transmitted infection; UKHSA, UK Health Security Agency.

Since 2012, there have been major changes in sexual health policy and service delivery in England (figure 1). These include reforms to public health provision and commissioning, expansion of targeted and pathogen-specific prevention programmes and evolving health equity approaches. Key changes are summarised below.

### Structural reforms in public health and commissioning

The 2012 Health and Social Care Act transferred responsibility for public health from the National Health Service (NHS) to local government,^13^ creating fragmented commissioning across local authorities, clinical commissioning groups and NHS England. Ambiguity over commissioning responsibilities was highlighted by early debates on whether funding for HIV pre-exposure prophylaxis (PrEP) should lie with local authority public health budgets or NHS England HIV treatment budgets,^14^ with NHS England ultimately assuming responsibility.

As funding pressures intensified, SHSs increasingly shifted toward online and postal self-sampling models at the expense of face-to-face provision, potentially exacerbating inequalities for vulnerable groups, including those experiencing digital exclusion, low health literacy or difficulties with self-sampling.^15 16^ Subsequent reforms under the 2022 Health and Care Act sought to address commissioning fragmentation by establishing integrated care systems and reducing reliance on competitive retendering.^17^ However, disbanding of NHS England in March 2025 introduced further uncertainty around governance and commissioning structures.

NHS cost recovery strategies in 2015 introduced charging overseas visitors for some healthcare services. Although sexual health and HIV care services remain exempt,^18^ it is unclear whether broader charging policies might indirectly affect access to and continuity of sexual healthcare, especially in vulnerable or marginalised groups.

### Reconfiguration and digitalisation of sexual health services

The COVID-19 pandemic and social restrictions imposed through 2020–2021, followed by the mpox outbreak in 2022 catalysed further reconfiguration of sexual healthcare delivery, accelerating adoption of digital and remote service models. Emerging evidence continues to explore uptake inequalities and accessibility of online services.^16^

### Progress and adaptation in key prevention programmes

The National Chlamydia Screening Programme (NCSP)^11^ shifted focus in 2021 towards detecting untreated infections in young women, deprioritising routine screening in men, reflecting long-standing recognition that the programme was unlikely to reduce population-level chlamydia prevalence. Uncertainty remains around adherence to new guidance, unintended consequences and impact on reducing reproductive harm.

Following the WHO’s 2008 Cervical Cancer Elimination Initiative,^19^ the UK introduced human papillomavirus (HPV) vaccination for adolescent girls, with catch-up for older women. The programme shifted from bivalent to quadrivalent vaccine and nonavalent vaccines and from three doses to one dose in 2023. Uptake has exceeded 80%, though coverage declined during the COVID-19 pandemic and has yet to fully recover.^20^ Evidence shows reductions in the prevalence of cancer-causing HPV types, genital warts and cervical cancer incidence in women.^21^ Ensuring equitable vaccination access and cervical cancer screening and identifying groups at risk are critical to achieving cervical cancer elimination in England by 2040.^22^

HPV vaccination for GBMSM was piloted in SHSs in 2016 and offered opportunistically from 2019; the same year, routine vaccination was extended to all adolescent boys. The impact of HPV vaccination on penile and other cancers among GBMSM will be carefully monitored in coming years.

### HIV and viral hepatitis elimination

National HIV surveillance in England showed rising new HIV diagnoses from 2024 following periods of declining or stable trends in GBMSM and heterosexual groups.^23^ Declines in HIV incidence from 2012 to 2023, particularly among GBMSM, reflect the success of scaled-up combination prevention, including treatment as prevention,^24^ regular HIV testing and increased access to PrEP. However, prevention gains have been less pronounced in heterosexual groups.^23 25^ By 2023, over 86 000 SHS attendees,^23^ mostly GBMSM, had accessed PrEP following routine commissioning after the 2017–2020 PrEP Impact Trial.

The 2022–2025 HIV Action Plan^26^ aligned national efforts towards HIV elimination with WHO 2030 elimination targets, including equity measures for preventative intervention uptake and stigma reduction. From 2023, opt-out BBV testing in emergency departments (ED) was implemented following pilots^27^ in areas of high or extremely high HIV prevalence (≥2 per 1000 people aged 15–59 years), alongside recommended HIV testing at general practice registration.^28^ In alignment with the WHO’s 2030 viral hepatitis elimination targets,^29^ hepatitis B and hepatitis C testing is now integrated into opt-out ED testing. Effective monitoring will be essential to evaluate progress towards elimination.

### Targeted prevention interventions for GBMSM

GBMSM continue to experience a disproportionate burden of STIs, accounting for roughly half of all new HIV, gonorrhoea and infectious syphilis diagnoses in England in 2023, despite representing less than 5% of the male population.^30 31^ Rising bacterial STI diagnoses in this group over the past two decades^5^ highlight the need for better prevention strategies, though surveillance in 2024 suggests a break in increasing chlamydia and gonorrhoea diagnosis trends.^3^

In 2023, the Joint Committee on Vaccination and Immunisation (JCVI) recommended targeted meningococcal B (4CMenB) vaccination for the prevention of gonorrhoea in GBMSM and other individuals at high-risk based on evidence of vaccine-effectiveness and cost-effectiveness,^32^ with roll-out in August 2025.^33^ GBMSM are also opportunistically offered vaccination for hepatitis A, hepatitis B and since 2019, HPV (following piloting starting in 2016) in SHSs, though uptake was disrupted during COVID-19.^30 34^

Following a multicountry outbreak of mpox clade IIb first identified in the UK in May 2022,^35^ an mpox vaccination programme, using smallpox vaccine,^36^ was rapidly implemented for GBMSM and other groups at risk. In 2023, the JCVI recommended routine opportunistic vaccination through SHSs which began in August 2025.^37 38^

Self-sourcing of doxycycline postexposure prophylaxis (doxyPEP) for the prevention of STIs has been reported by GBMSM in the UK from as early as 2019.^39^ UK-specific guidelines now recommend its use for individuals at high-risk of STIs primarily for syphilis prevention,^40^ aligning with some international policy updates.^41–44^ Routine asymptomatic screening for GBMSM, including those using PrEP, remains under review amid concerns about antibiotic overuse. Cost-effectiveness evidence is needed to inform future recommendations.^45 46^ As a relatively small population with a disproportionately high burden of STIs, HIV and experienced stigma, GBMSM require continued surveillance.^3^

### Young people and sex education

Young people remain at high-risk of bacterial STIs. In England, gonorrhoea diagnoses among those aged 15–24 rebounded in 2021 after COVID-19 social restrictions were lifted and continued to rise through 2023,^30 47^ highlighting ongoing vulnerability and need for sustained and targeted interventions. In 2020, the Department for Education introduced compulsory relationships and sex health education for secondary schools in England to strengthen understanding of relationships, sexual health and well-being.^48^ Comprehensive evaluations of this policy and its delivery are needed to assess longer-term impacts on sexual health and well-being.

### Health equity

Efforts to improve sexual health have increasingly focused on health equity. The 2022 Women’s Health Strategy for England outlined actions to improve sexual and reproductive health, including integrating essential women’s services with sexual healthcare to remove structural barriers and improve health outcomes.^49^ In parallel, the 2023 UKHSA Health Equity Strategy recommended enhanced data collection, inclusive service design and community engagement to address health inequalities.^50^ The British Association for Sexual Health and HIV also highlighted the need for more inclusive health promotion, including transgender and gender-diverse inclusive SHS-design.^51^

### Blood donation policy reform

In 2021, a revised blood donor selection policy was introduced following review by the ‘For the Assessment of Individualised Risk’ steering group.^52^ Blanket restrictions preventing men who disclosed same-sex partners from donating blood were replaced with an individualised risk assessment of recent sexual behaviour and safe-sex practices. Ongoing monitoring is needed to ensure donor diversity and blood safety.

## Sexual health data resources

This section focuses on key STI and reproductive health data sources. Comprehensive detail of individual sources are in online supplemental appendix I. While the focus is on England, some sources span the UK. Private-sector HIV and STI testing data are excluded.

10.1136/sextrans-2025-056754.supp1 Supplementary data

### National and targeted surveys

The National Surveys of Sexual Attitudes and Lifestyles (Natsal) are large, decennial, cross-sectional household surveys that have collected data on sexual behaviour, service use and sexual health in Britain (England, Scotland and Wales) since 1990. They provide nationally representative self-reported behavioural and health data not available elsewhere, alongside biological sampling to estimate population-level prevalence of key STIs and markers of antimicrobial resistance (AMR). Natsal-4, completed in 2024, introduced consent for data linkage to health data (eg, cervical screening) and administrative datasets (eg, Office for National Statistics (ONS) Census, National Pupil Database), enabling richer cross-sectional analyses. Natsal surveys are essential for assessing population need, including among those not accessing services, and its probability sampling supports national generalisability. However, the decennial design creates gaps in data availability, and some key populations are under-represented or excluded either due to small numbers in the general population (eg, GBMSM), stigma (eg, sex workers) or design features that omit certain groups (eg, prisoners, people experiencing homelessness).

Other general health surveys, including the Health Survey for England and the English Longitudinal Study of Ageing, occasionally include modules on sexual behaviours and STIs.^53 54^ Targeted behavioural surveys, such as the ‘Reducing inequalities in Sexual Health’ (RiiSH) surveys, the Gay Men’s Sexual Health Surveys (GMSHS) and the Women’s Reproductive Health Survey, provide periodic insights into SHS use, intervention uptake and well-being among key populations. By design, these surveys usually rely on convenience sampling to achieve sufficient sample sizes among typically underserved populations of interest.

### National surveillance of STIs, HIV and reproductive health

National STI and HIV surveillance in England draws on multiple sources including SHSs and laboratory-based sentinel surveillance covering nationally commissioned services provided through the NHS, and targeted, bio-behavioural monitoring. The GUMCAD STI Surveillance System (GUMCAD), implemented in 2008, is the backbone of STI surveillance, collecting electronic disaggregated data from all commissioned NHS SHSs in England, replacing paper-based KC60 returns. Recent enhancements have expanded GUMCAD to include behavioural data and gender identity.^55^ GUMCAD supports longitudinal analyses within the same clinics as cross-tracking across services remains limited due to the use of clinic-specific pseudoanonymised patient identifiers which cannot be linked.

Sentinel surveillance collects clinical information through a network of reporting sites selected to be representative of the wider population. While national STI surveillance provides comprehensive data at population-level, sentinel surveillance systems such as the Gonococcal Resistance to Antimicrobials Surveillance Programme (GRASP) and Mycoplasma genitalium Antimicrobial Resistance Surveillance (MARS) complement national STI surveillance by providing annual data on antimicrobial susceptibility and resistance among key populations, such as GBMSM. These systems are essential for monitoring the emergence and spread of AMR.

The HIV and AIDS reporting system (HARS) and other national disaggregate datasets provide detailed longitudinal data on people living with HIV and accessing care. Together, these systems support analyses of service uptake, clinical outcomes and preventative intervention evaluation.

Reproductive health surveillance captures activity related to antenatal infectious disease screening, abortions, conceptions and contraception uptake among nationally commissioned services. Key sources include the Infectious Diseases in Pregnancy Screening Programme, ONS Abortion and Conceptions statistics, Sexual and Reproductive Health Activity Data Set, Maternity Services Dataset and Female Genital Mutilation Enhanced Dataset.

### Data from general practice and hospital services

Sexual healthcare, including STI and HIV testing, is increasingly offered outside specialist services through general practice (family doctor) and hospital settings. Integration of this service data into routine surveillance and evaluation is crucial to avoid national-level gaps. Primary care data sources, such as the Clinical Practice Research Database, provide longitudinal, individual-level data linked via NHS number (ie, unique patient identifier), including demographics, patterns of service use, health behaviours and referrals to secondary care that can be used for analyses of care pathways and clinical outcomes. However, data complexity, coverage and data quality varies regionally. Secondary care datasets, notably the Hospital Episodes Statistics (HES), record admissions and attendance reasons, enabling identification of STI-related and reproductive health-related sequelae and complications. HES data are widely used for service commissioning and generally consistent in data coding and completeness.

### Administrative, census and registry data

Administrative data sources and population registries offer broader insights across population, education and health domains. The decennial ONS Census provides detailed national data on evolving individual and household characteristics that guide public funding allocations. In 2021, sexual orientation and gender identity were included for the first time, improving the understanding of the size and distribution of key populations (eg, transgender groups).^31^ However, concerns remain about reporting reliability with further evaluation needed.^56^

Cancer is a rare but serious consequence of some STIs, including HPV and viral hepatitis. Cancer registries track diagnoses and outcomes, playing an important role in cervical cancer surveillance. Similarly, for some rare infections like hepatitis C, national disease registries are used to quantify burden and monitor elimination efforts.

### Other research and biobank resource

There is a growing body of SHS research^57–59^ and biobanking resources (ie, UK Biobank) that complement routine data, offering enhanced information of service utilisation and biological, health and lifestyle information.

## Discussion

### Working towards data source synergies using data integration approaches

The sexual health policy landscape in England has undergone major and frequent changes from 2012 to 2024, creating a need to evaluate whether policies are achieving their intended impacts. There are a wealth of data sources in England and the UK (online supplemental appendix I), each providing unique but often incomplete insights. All have limitations in scope, representativeness or methodology. We propose that by carefully selecting complementary datasets for specific research, policy or evaluation questions and using data integration, it is possible to combine their respective strengths and offset individual limitations to provide a more comprehensive picture of sexual health at a population-level.

Effective data integration faces several challenges and can be constrained by data access, privacy and ethical considerations, data quality and standardisation, and technical complexities (box 1). Overcoming context-specific challenges is essential to maximise the value of integrated data for research, monitoring and evaluation. Crucially, meaningful community buy-in is essential for ensuring legitimacy, maintaining trust and facilitating sustained collaboration.

Box 1 **Key challenges to data integration**

**Partnership and engagement**

- Community partnership and engagement to ensure legitimacy, trust and enable meaningful, sustained collaboration.

**Access, governance and regulation**

- Data access barriers, requiring ethical and governance approvals.
- Complex governance and regulatory requirements, taking time and resources.
- Ensuring appropriate consent for secondary use and data linkage.

**Privacy and ethical considerations**

- Privacy and confidentiality concerns, especially for rare outcomes or minority groups where deductive disclosure might be a risk.

**Data structure, quality and standardisation**

- Data quality and standardisation, requiring meticulous harmonisation to ensure comparability.
- Lack of shared identifiers across data sources.

**Technical and methodological complexity**

- Integration methods, requiring statistical and data management expertise.
- Practical implementation, requiring expertise, time, resources, approvals and computational power.
- Time lags and data refresh cycles, requiring persistence, coordination and long-term funding.

**Analysis limitations**

- Potential misinterpretation of variables and data structure, requiring subject experts to guide integration and interpretation.
- Risk of ecological fallacy where reliance on aggregate data can obscure individual-level variation, potentially leading to incorrect inferences.
- Surveillance bias that may overrepresent groups or conditions that may reinforce stigma if not critically examined, requiring cautious interpretation.

Integration approaches should be matched to the research, policy or evaluation question. For example, individual-level linkage can support assessments of care pathways and outcomes but is often limited by governance requirements, pseudonymisation and public trust concerns. Where feasible, these can be addressed through appropriate governance arrangements, trusted research environments and transparent public engagement. On the other hand, aggregate-level integration of surveillance and survey data might provide a more pragmatic and accessible option, particularly for assessing reach and equity but does not allow detailed analysis, for example of subgroups, and can be prone to ecological fallacy if group associations do not apply at individual-level. In some cases, mathematical modelling can synthesise multiple data sources.^60^

### Data source integration in practice

We provide three high-level conceptual exemplars illustrating how data integration approaches might work in practice.

### Evaluating the impact of population-level sexual health interventions

Several data sources support evaluation of population-level sexual health interventions. These include national STI surveillance, primary and secondary care data, cancer screening and outcome registries, and nationally representative surveys that capture information on preventative uptake and outcomes. Each source has gaps; surveillance reflects service users, surveys offer broader population-level or targeted insights, and linked care data reveal care pathways beyond traditional settings, supporting assessment of disease trends, STI sequelae and preventative care across diverse population groups.

**Figure 2:**
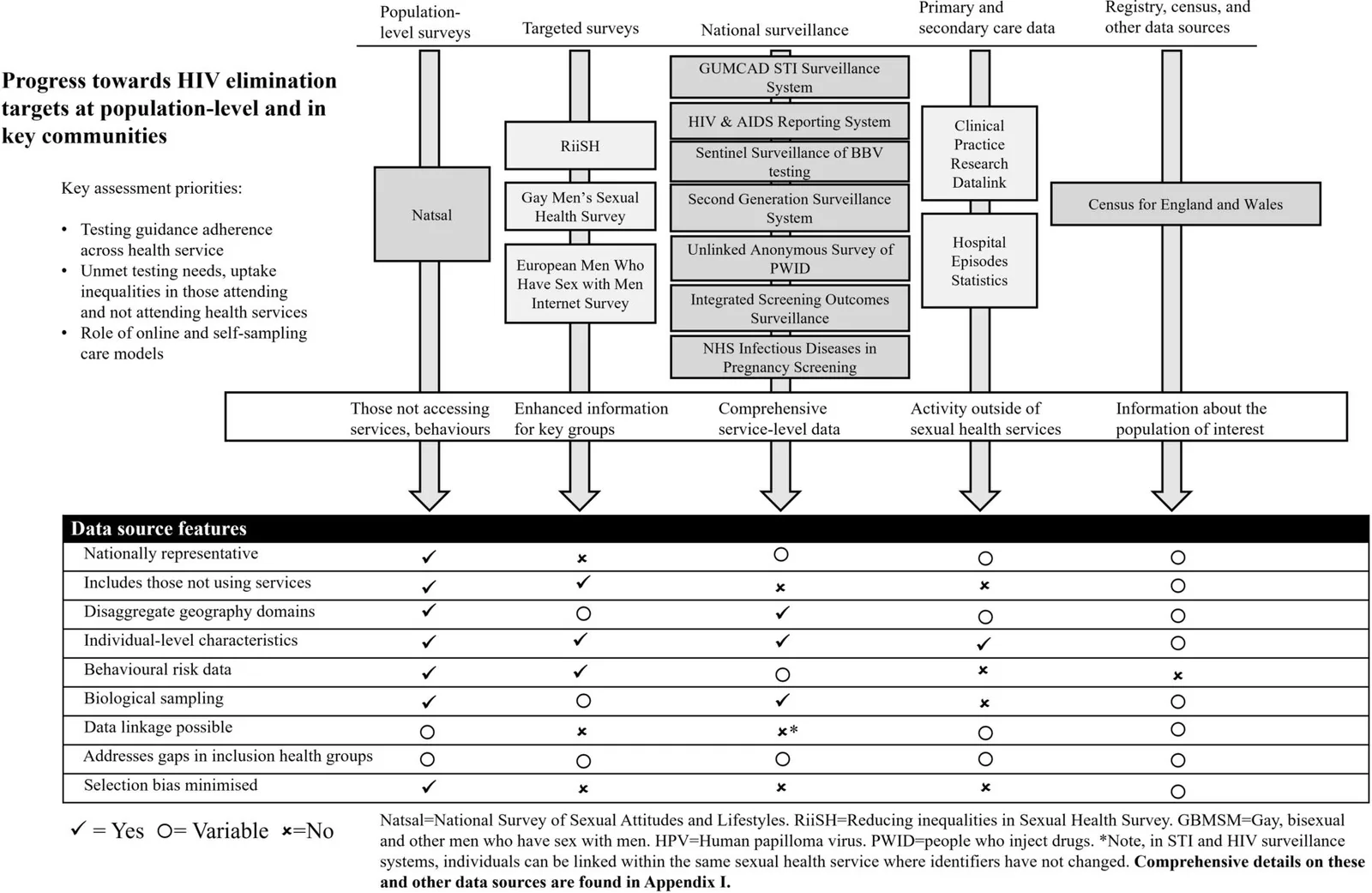
Data integration exemplar showing how to use data source synergies to assess the impact of population-level sexual health interventions and progress towards HIV elimination. BBV, blood-borne virus; GUMCAD, GUMCAD STI Surveillance System;NHS, National Health Service; STI, sexually transmitted infection.

For example, progress towards HIV elimination in England can be assessed by integrating nationally representative behavioural surveys (eg, Natsal), national STI/HIV surveillance (eg, GUMCAD/HARS) and targeted surveys (eg, RiiSH) (figure 2). Natsal provides population-level data on behaviours and testing (including those not accessing services), enabling assessment of testing coverage and unmet need; national surveillance offers detailed service-level and individual-level data on testing activity, diagnoses and linkage to care; and targeted surveys such as RiiSH focus on key populations, such as GBMSM. Integrating these complementary sources mitigates the limitations of individual datasets, allowing examination of unmet HIV testing needs among service users and non-users, the role of online and postal-based self-sampling, and inequalities in testing uptake across populations and services. Such approaches help capture groups missed by national surveillance and other data sources, crucial to informing selection bias, modelling and cost-effectiveness assessments. Similar strategies can evaluate HPV vaccination impact and adherence to updated NCSP recommendations.

### Understanding behavioural drivers of STI and HIV trends

Research surveys remain the main source of behavioural information that contextualise, and in some cases, explain STI and HIV diagnosis trends. Surveys provide insights into changing sexual risk behaviours, partnership dynamics and uptake or unmet need of preventative interventions, such as condom use or PrEP adoption among key populations. However, isolated assessments can fail to capture the full complexity of behavioural trends, particularly when conducted infrequently or where they may lack sufficient participation of key populations, potentially under-representing emerging patterns in groups at risk or those not sampled at all.

These challenges can be addressed through integrated approaches that draw on multiple sources of behavioural data, both from the general population (eg, Natsal) and from groups most at risk (eg, RiiSH, GMSHS) which in turn helps with interpretation of surveillance data (eg, GUMCAD). This approach uses behavioural and clinical data to detect emergent risks at population-level and within key populations, enabling improved monitoring and risk-assessment, and supporting targeted and effective public health responses (eg, estimating GBMSM eligible for mpox vaccination and 4CMenB vaccination for the prevention of gonorrhoea).

### Updating population prevalence and characterising antimicrobial resistance

Population-level prevalence data for STIs and HIV are limited, as most surveillance focuses on SHS users. As a result, infections among asymptomatic people, those not accessing care or marginalised groups less likely to engage with health systems are often missed. This introduces significant bias to estimates of disease burden, limiting understanding of population-level transmission dynamics.

Natsal surveys help to fill this gap by providing prevalence and distribution estimates using self-report measures and biosampling (eg, *Chlamydia trachomatis, Neisseria gonorrhoeae, Mycoplasma genitalium*, *Trichomonas vaginalis*, HPV types 16/18, HIV antibodies).^60–62^ However, infrequent surveys limit their ability to assess emerging trends following, for example, guideline and policy changes. Natsal estimates, together with complementary data sources (eg, GUMCAD, HARS, GMSHS) can support mathematical modelling of STI/HIV transmission and associated outcomes, estimate diagnosed and undiagnosed prevalence using multiparameter synthesis methodologies,^63^ and evaluate the impact of interventions such as PrEP or doxyPEP. Integrated approaches using microbial genomics data could be used to examine markers of AMR at a population-level (eg, Natsal) and in key populations like GBMSM or ethnic minority groups (eg, GRASP, MARS) to inform clinical management and treatment guidelines.^62 64^

## Conclusion

This review makes the conceptual case for data synergy, encouraging sexual health researchers and policymakers to critically reflect on use of integration approaches in their own contexts. The past decade has seen changes and frequent policy shifts in an environment of changing STI and HIV epidemiology and expanding service digitalisation. Evidence-based policy making demands robust evaluation of such policies. Ultimately, integrated approaches can maximise the return on investment of these mostly publicly funded data sources, enhancing our understanding of evolving population-level needs, disparities in access, and broader social and service-related contexts influencing sexual health epidemiology.
