# Supplementary material for: From silos to synergies: harnessing the power of sexual health data sources in England to understand changing epidemiology and inform policy
Source: Sex Transm Infect. 2026 Jun 8;102(6):401–8. doi: 10.1136/sextrans-2025-056754 (PMC13539839; doi:10.1136/sextrans-2025-056754)
Supplement: Supplementary file 1 [file sextrans-102-6-s001.pdf]

Appendix I. Sexual health data sources

This appendix presents a selection of key sexual health data sources. Notably, data from private STI and HIV testing services (i.e. direct to consumer tests) are not included in this resource. The information provided reflects the status of data holders at time of writing. Data sources held by NHS England may be subject to change.

Sections:

- **Survey data sources**
  - **Population-level sexual health**
  - **Targeted**
  - **General health**
- **National surveillance of STIs and HIV**
- **Reproductive health data sources, including surveillance***
- **Primary and secondary care data sources**
- **Census, registries, additional administrative data**
- **Other data sources**

*Note, this not an exhaustive list of all reproductive health data sources given sexual health scope.

**Data source (Data holder)**	**Description**	**Main strengths**	**Main limitations**
**Survey data sources**			
**Population-level sexual health**			
**National Surveys of Sexual Attitudes and Lifestyles (Natsal)[1-6]**<br><br>(University College London [UCL], London School of Hygiene and Tropical Medicine [LSHTM], University of Glasgow, National Centre for Social Research [Natcen)])<br><br>*Note, Natsal funded by Wellcome Trust, Economic and Social Research Council (ESRC) and National Institute for Health and Care Research (NIHR)	Serial, decennial cross-sectional probability survey collecting self-reported data on sexual behaviours, sexual and reproductive health outcomes, and attitudes alongside detailed sociodemographic data and biosamples in the general population in Britain	▪ Includes expansive demographic, lifestyle, health behaviour information representative of the general population<br>▪ Includes individuals not accessing healthcare services<br>▪ Comparability of core questions over time and between birth cohorts<br>▪ Includes collection of biological samples<br>▪ Can link to other health, administrative, education data sources (e.g. National Pupil database, NHS Cervical Screening Programme)<br>▪ Expanded gender identity questions<br>▪ Use of computer assisted self-interviewing for the most sensitive questions to limit some reporting and social desirability bias<br>▪ Surveys archived in UK Data Service	▪ Low numbers of inclusion health populations<br>▪ Cross-sectional data collection; sampling, recall, social desirability biases<br>▪ Declining survey participation<br>▪ Timeliness (conducted only once every 10 years)<br>▪ Lack of generalisability to individuals outside of private households
**Targeted surveys**			
**Reducing inequalities in Sexual Health (RiiSH) Survey[7 8]**	Serial, cross-sectional online survey assessing the sexual health and well-being of gay,	▪ Includes expansive demographic, lifestyle, health behaviour information	▪ Convenience sample

(UCL/UK Health Security Agency [UKHSA])	bisexual, and other men who have sex with men in the UK	▪ Online self-completion, pseudo-anonymity to limit some reporting and social desirability bias<br>▪ Targeted monitoring of key population<br>▪ Includes individuals not utilising sexual health services<br>▪ Annual reporting, comparability of core questions over time<br>▪ Expanded gender identity questions	▪ Cross-sectional data collection; sampling, recall, social desirability biases, digital exclusion<br>▪ Generalisability to wider targeted population<br>▪ Cannot establish causality between behavioral data and outcomes of interest<br>▪ No linkage possible
**Gay Men's Sexual Health Survey (GMSHS)**[9-11]<br><br>(UCL/UKHSA)	Serial, cross-sectional bio-behavioural survey assessing sexual health of gay, bisexual, and other men who have sex with men recruited from London social venues (i.e., pubs, clubs, bars, sex-on-premises venues)	▪ Longitudinal comparability of core questions<br>▪ Consistent methodology since 1996<br>▪ Targeted monitoring of key population<br>▪ Includes individuals not utilising sexual health services<br>▪ Oral fluid sampling (HIV antibody testing)<br>▪ Question modules informed by community stakeholders<br>▪ Expanded gender identity questions	▪ Convenience sample<br>▪ Sampling, recall, social desirability biases<br>▪ Establishing temporality<br>▪ Generalisability<br>▪ Timeliness, reporting every 5 years<br>▪
**European Men who have sex with men Internet Survey (EMIS)**[12]<br><br>(LSHTM/Sigma Research)	Serial, cross-sectional online survey assessing the sexual health and well-being of gay, bisexual, and other men who have sex with men in Europe (includes the UK)	▪ Includes expansive demographic, lifestyle, health behaviour information<br>▪ Multi-country, multi-language, launched cross 50 countries<br>▪ Online self-completion, pseudo-anonymity to limit some reporting and social desirability bias<br>▪ Targeted monitoring of key population<br>▪ Decennial reporting (approximately), comparability of core questions over time	▪ Convenience sample<br>▪ Cross-sectional data collection; sampling, recall, social desirability biases, digital exclusion<br>▪ Generalisability to wider targeted population<br>▪ Cannot establish causality between behavioral data and outcomes of interest<br>▪ No linkage possible
**Reproductive Health Survey for England**[13]<br><br>(Department for Health and Social Care [DHSC])/LSHTM)	Cross-sectional survey assessing reproductive health experiences of women and people assigned female at birth (latest data collection in 2023)	▪ Includes expansive demographic, lifestyle, health behaviour information<br>▪ Largest reproductive health survey in England<br>▪ Includes information on periods and menopause, preventing pregnancy and trying to get pregnant, pregnancy and birth experiences, and reproductive health conditions<br>▪ Online self-completion, pseudo-anonymity to limit some reporting and social desirability bias	▪ Convenience sample<br>▪ Cross-sectional data collection; sampling, recall, social desirability biases, digital exclusion<br>▪ Generalisability to wider targeted population<br>▪ Cannot establish causality between behavioral data and outcomes of interest<br>▪ No linkage possible
**Positive Voices Survey**[14]<br><br>(UKHSA)	Serial, cross-sectional survey of people living with HIV	▪ Nationally representative<br>▪ Includes expansive demographic, lifestyle, health behaviour information<br>▪ Online and paper-based self-completion, pseudo-anonymity to limit some reporting and social desirability bias<br>▪ Captures stigma related metrics	▪ Cross-sectional data collection; sampling, recall, social desirability biases, digital exclusion<br>▪ Declining survey participation<br>▪ Restricted to service users, no data on those not accessing care

Ogaz D, *et al. Sex Transm Infect* 2026; 102:401–408. doi: 10.1136/sextrans-2025-056754

		▪ 5-yearly reporting (approximately), comparability of core questions over time<br>▪ Some linkage possible to surveillance data sources<br>▪ Expanded gender identity questions	
**PrEP Users Survey**[15]<br><br>(UKHSA)	Cross-sectional survey of individuals who tried to access HIV PrEP	▪ Includes expansive demographic, lifestyle, health behaviour information<br>▪ Large sample of key population using who tried to access HIV PrEP<br>▪ Online self-completion, pseudo-anonymity to limit some reporting and social desirability bias<br>▪ Captures stigma related metrics<br>▪ Survey samples prior to and following UK commissioning of HIV PrEP in sexual health services<br>▪ Expanded gender identity questions	▪ Convenience sample<br>▪ Cross-sectional data collection; sampling, recall, social desirability biases, digital exclusion<br>▪ Generalisability to wider targeted population<br>▪ Cannot establish causality between behavioral data and outcomes of interest<br>▪ Timeliness, 5-yearly reporting (approximately), comparability of core questions over time<br>▪ No linkage possible
**General health surveys**			
**Health Survey for England**[16]<br><br>(NHS England)<br><br>Equivalent surveys for Scotland, Northern Ireland, Wales:<br><br>▪ Scottish Health Survey (Scottish Government)[17]<br>▪ Health Survey Northern Ireland (Department of Health NI)[18]<br>▪ National Survey for Wales (Welsh Government)[19]	Serial, cross-sectional surveys assessing trends in the health, and health related behaviours, of adults and children; includes biological sampling	▪ Generalisable sample of private households, uses national weighting<br>▪ Includes some demographic, lifestyle, health behaviour information<br>▪ Includes some anthropomorphic measurements<br>▪ Comparability of core questions over time<br>▪ Regular/annual reporting<br>▪ Supports public health policy, surveillance, and epidemiological research	▪ Generally limited sexual behaviour information, but periodic inclusion of related questions<br>▪ Cross-sectional data collection; sampling, recall, social desirability biases<br>▪ Lack of generalisability to individuals outside of private households<br>▪ No linkage possible
**The General Practitioner Patient Survey (GPPS)**[20]<br><br>(NHS England)	Cross-sectional survey assessing individual-level experiences of healthcare services provided by GP practices in England	▪ Includes information on service access and quality of care in general practice<br>▪ Includes some demographic, lifestyle information<br>▪ Annual reporting (approximately), comparability of core questions over time<br>▪ Online self-completion, pseudo-anonymity to limit some reporting and social desirability bias	▪ Limited sexual behaviour information<br>▪ Cross-sectional data collection; sampling, recall, social desirability biases, digital exclusion<br>▪ Generalisability to wider targeted population<br>▪ Cannot establish causality between behavioral data and outcomes of interest<br>▪ No linkage possible
National surveillance of STIs & HIV			
**GUMCAD STI Surveillance System**[21-23]<br><br>(UKHSA)	Mandatory pseudonymised disaggregate dataset collecting clinical activity of all specialist and other commissioned non-specialist sexual health services in England	▪ Coverage for all local authority commissioned sexual health services<br>▪ High granularity with patient-level, attendance-based data; includes some demographic information	▪ Limited sexual behaviour information (however, expanded following piloting)<br>▪ No individual-level tracking across services (but evidence suggests movement between services is uncommon)

		▪ Quarterly reporting, comparability of service activity and diagnosis trends at population-level over time<br>▪ Includes individual-level, longitudinal data (can build cohort studies if use consistent service)<br>▪ Expanded gender identity questions	▪ Some data quality limitations with introduction or updates to coding (periodic)<br>▪ Limited to reporting of pre-defined activity and diagnosis codes (however, periodically reviewed)<br>▪ Cannot link data to external data sources (outside of select surveillance data sources)<br>▪ Restricted to service users, no data on those not accessing care
**CTAD Chlamydia Surveillance System**[24]<br><br>(UKHSA)	Mandatory pseudonymised disaggregate dataset collecting chlamydia testing and diagnosis data from all laboratories commissioned by local authorities or the NHS to carry out chlamydia testing	▪ Coverage from all laboratories conducting commissioned chlamydia testing, which includes testing outside of sexual health services (e.g., general practice)<br>▪ Includes some demographic information<br>▪ Quarterly reporting, comparability of testing activity and diagnosis trends at population-level over time<br>▪ Includes individual-level, longitudinal data (can build cohort studies if use consistent service)<br>▪ Supports evaluation of National Chlamydia Screening Programme (NCSP)	▪ Limited sexual behaviour information<br>▪ No individual-level tracking across services (but evidence suggests movement between services is uncommon)<br>▪ Some data quality limitations with introduction or updates to coding (periodic)<br>▪ Limited to reporting of pre-defined activity and diagnosis codes (however, periodically reviewed)<br>▪ No gender identity, sexual orientation indicators (unless linked to select surveillance data sources)<br>▪ Cannot link data to external data sources (outside of select surveillance data sources)<br>▪ Restricted to service users, no data on those not accessing care
**HIV and AIDS reporting system (HARS), Survey of Prevalent HIV Infections Diagnosed (SOPHID), HIV and AIDS New Diagnoses Database (HANDD), Recent Infection Testing Algorithm (RITA), CD4 Surveillance**[25]<br><br>(UKHSA)	Disaggregate dataset on all HIV diagnoses, associated clinical history and HIV care attendances in England	▪ Coverage for all commissioned services of HIV care<br>▪ Includes some demographic information<br>▪ Quarterly reporting, comparability of diagnosis setting and diagnosis trends at population-level over time<br>▪ Includes individual-level, longitudinal data (can build cohort studies)<br>▪ Expanded gender identity questions	▪ Does not capture undiagnosed HIV infections<br>▪ Limited sexual behaviour information<br>▪ Cannot link data to external data sources (outside of select surveillance data sources)<br>▪ Restricted to service users, no data on those not accessing care
**Sentinel Surveillance of Bloodborne Virus testing in England (SSBBV)**[26]<br><br>(UKHSA)	Sub-national laboratory-based surveillance system for blood-borne virus (BBV) testing activity and results in England	▪ Data on all positive and negative BBV tests taken in community, primary and secondary care services that are processed by sentinel NHS laboratories (enabling testing coverage and positivity)<br>▪ Wide testing coverage represents general practice registered population in England; includes testing in other diverse settings (e.g. prisons, drug services, hospitals)	▪ Limited sexual behaviour information<br>▪ Limited sociodemographics (unless linked to select surveillance data sources)<br>▪ Coverage variability, shifts in geographical representation<br>▪ Laboratory participation in SSBBV is voluntary (~40% laboratory coverage, however full London laboratory representation)<br>▪ Variable data quality for linkage and de-duplication

Ogaz D, *et al. Sex Transm Infect* 2026; 102:401–408. doi: 10.1136/sextrans-2025-056754

		▪ Quarterly reporting, comparability of testing activity and diagnosis trends at sub-national level over time<br>▪ Includes individual-level, longitudinal data (can build cohort studies)<br>▪ Supports trend analysis, inequality monitoring, and modelling for HIV and blood borne virus elimination targets	▪ Restricted to service users, no data on those not accessing care
**Second Generation Surveillance System (SGSS)[27]**<br><br>(UKHSA)	National laboratory reporting system used in England to capture reports of notifiable infectious diseases and antimicrobial resistance	▪ Main system through which NHS diagnostic laboratories submit mandatory reports, broad coverage of diagnostic laboratories in England<br>▪ Includes some demographic information<br>▪ Quarterly reporting (across several surveillance and pathogen specific reporting streams), comparability of core metrics over time<br>▪ Some linkage capability (to select surveillance data sources)	▪ Limited sexual behaviour information<br>▪ Variable data quality for linkage (to select surveillance data sources) and de-duplication<br>▪ Reporting of STIs is voluntary and incomplete<br>▪ Data reporting lag and may lack standardisation in laboratory coding practices<br>▪ Restricted to service users, no data on those not accessing care
**Gonococcal resistance to antimicrobials surveillance programme (GRASP)[28]**<br><br>**Mycoplasma genitalium antimicrobial resistance surveillance (MARS)[29]**<br><br>(UKHSA)	National sentinel surveillance system collecting gonococcal isolates to detect and monitor antimicrobial resistance in *Neisseria gonorrhoeae* and potential treatment failures<br><br>National sentinel surveillance system collecting isolates to detect and monitor antimicrobial resistance in Mycoplasma genitalium and potential treatment failures	▪ Isolates collected from testing undertaken in primary diagnostic laboratories and linked pseudonymously to GUMCAD and enhanced questionnaires for supplemental demographic and clinical information<br>▪ Annual reporting of antimicrobial susceptibility trends, comparability of core metrics over time<br>▪ Includes individual-level, longitudinal data (can build cohort studies)<br>▪ Regular review of sampled population	▪ No sexual behaviour data (unless linked to select surveillance data sources)<br>▪ Limited sociodemographics (unless linked to select surveillance data sources)<br>▪ Cross-sectional and sentinel data collection<br>▪ Potential for sampling bias, generalisability of sentinel collection<br>▪ Restricted to service users, no data on those not accessing care
**UKHSA reference laboratories[5]**<br><br>**STI Reference Laboratory (STIRL)[5]**<br><br>**Modular Open Laboratory Information System (MOLIS)[5]**<br><br>**Viral reference department (VRD) (includes HPV surveillance)[5]**<br><br>(UKHSA)	National and international reference centre<br><br>STI diagnostic and antimicrobial susceptibility testing undertaken at STIRL available from the MOLIS<br><br>VRD receives clinical samples and viral isolates from public health departments, NHS and commercial laboratories across the UK, internationally for specialist testing, virus characterisation and susceptibility testing	▪ Central diagnostic references, laboratory information systems, data warehouses integral to surveillance workflows<br>▪ Support for outbreak investigations<br>▪ Some linkage capability (to select surveillance data sources)<br>▪ Comparability of core metrics over time	▪ May not include undetected, non-referred cases<br>▪ No sexual behaviour data (unless linked to select surveillance data sources)<br>▪ Limited sociodemographics (unless linked to select surveillance data sources)<br>▪ Limited clinical data collected through MOLIS<br>▪ Restricted to service users, no data on those not accessing care
**Gastro Data Warehouse (GDW)[5]**<br><br>(UKHSA)	Processes microbiological, genomic, and antimicrobial resistance data is for *Shigella spp.* isolates sent to the UKHSA Gastrointestinal Bacterial Reference Unit (GBRU) for confirmation and typing.	▪ Data warehouse integral to surveillance workflows<br>▪ Support for outbreak investigations<br>▪ Some linkage capability (to select surveillance assets)<br>▪ Collection of enhanced epidemiological information, including travel history	▪ May not include undetected, non-referred cases<br>▪ No sexual behaviour data (unless linked to select surveillance assets)<br>▪ Limited sociodemographics (unless linked to select surveillance assets)<br>▪ Restricted to service users, no data on those not accessing care

		▪ Comparability of core metrics (including viral typing) over time	
**Unlinked Anonymous Monitoring Survey in People Who Inject Drugs (UAM PWID)**[30]<br><br>(UKHSA)	Annual, cross-sectional bio-behavioural survey of PWID conducted across agencies that provide services to people who inject psychoactive drugs, that include medical services, needle-exchange programmes, and outreach	▪ Regular collection of behavioural information, including some sexual risk behaviours<br>▪ Includes some demographic information<br>▪ Annual reporting, comparability of core questions and biosampling (dried blood spot, Hepatitis B/C, HIV) over time<br>▪ Wide geographical representation	▪ Cross-sectional data collection; sampling, recall, social desirability biases<br>▪ Shifts in geographical representation<br>▪ No linkage possible to surveillance data sources (at time of writing)<br>▪ Restricted to service users, no data on those not accessing care
**UK Blood Donor Survey**[31]<br><br>(UKHSA, NHS Blood and Transplant [NHSBT])	Cross-sectional, unlinked anonymous surveillance system that tests residual blood samples from consenting blood donors across the UK for markers of blood-borne viruses (e.g. HIV, hepatitis B, hepatitis C) to monitor prevalence and trends in low-risk populations	▪ Large assessment of donor compliance<br>▪ Provides consistent, long-term data sources on blood borne virus prevalence in a low-risk population<br>▪ Collection of some behavioural information (adapted from National Surveys of Sexual Attitudes and Lifestyles [Natsal])<br>▪ Support validation of other surveillance data sources	▪ Cross-sectional data collection; sampling, recall, social desirability biases; blood donors not representative of general population<br>▪ Limited sexual behaviour information<br>▪ Generalisability to wider donor population<br>▪ Irregular data collection<br>▪ No linkage possible to surveillance data sources
**Reproductive health data sources, including surveillance**			
**Sexual and Reproductive Health Activity Dataset (SRHAD)**[32]<br><br>(NHS England)	Mandatory dataset on contraceptive activity taking place at dedicated sexual and reproductive health (SRH) services in England	▪ National coverage across community-based SRH services<br>▪ Captured trends in contraception use, including methods and types; includes some demographic information<br>▪ Annual reporting, comparability of core metrics over time<br>▪ Supports monitoring of access to and uptake of reproductive health services	▪ Excludes services provided in general practice and some integrated sexual health services<br>▪ Limited sexual behaviour information<br>▪ Some data quality limitations with introduction or updates to coding (periodic)<br>▪ Limited to reporting of pre-defined activity and diagnosis codes (however, periodically reviewed)<br>▪ Restricted to service users, no data on those not accessing care
**NHS Infectious Diseases in Pregnancy Screening (IDPS)**[33]<br><br>**Integrated Screening Outcomes Surveillance Service (ISOSS)**[33]<br><br>(UKHSA, NHS England)	National surveillance system in the UK that collects data on pregnant women living with HIV and their infants to monitor outcomes of antenatal screening, treatment uptake, and vertical transmission<br><br>A national NHS screening programme in England that offers all pregnant women screening for HIV, hepatitis B, and syphilis to reduce vertical transmission and ensure timely intervention; mandatory dataset on antenatal screening of infectious diseases in pregnancy	▪ Wide coverage across pregnancy services<br>▪ Includes some demographic information<br>▪ Annual reporting<br>▪ Comparability of core metrics over time<br>▪ Monitors vertical transmission; captures detailed pregnancy and treatment outcomes; supports elimination targets for mother-to-child transmission	▪ Focused only on pregnancies diagnosed via screening<br>▪ Screening uptake may vary locally; may miss late or unreported cases; relies on timely clinical reporting<br>▪ Limited sexual behaviour information<br>▪ Restricted to service users, no data on those not accessing care
**Conception statistics for England and Wales**[34]<br><br>(Office for National Statistics, [ONS])	Annual statistics on conceptions to residents of England and Wales compiled from statutory notifications and surveys	▪ Population-level estimates<br>▪ Includes some demographic information<br>▪ Uses records of birth registrations collected under the Births and Deaths Registration Act 1953 and of abortion	▪ Aggregated data, limiting detailed individual-level analyses<br>▪ Limited sexual behaviour information<br>▪ Conception statistics are estimates<br>▪ Based on estimates of service users

		notifications supplied under the Abortion Act 1967<br>▪ Local data availability for select indicators<br>▪ Annual reporting, comparability of core metrics over time	▪ Do not include miscarriages or illegal abortions (though illegal abortions likely rare in UK)
**Abortion statistics for England and Wales**[35]<br><br>(DHSC)	Annual statistics on abortions to residents of England and Wales collected from all registered abortion providers, detailing the number, timing, method, and demographic characteristics of abortions performed	▪ Population-level estimates with standardised reporting<br>▪ Includes some demographic information<br>▪ Uses records of abortion notifications supplied under the Abortion Act 1967<br>▪ Local data availability for select indicators<br>▪ Annual reporting, comparability of core metrics over time	▪ Aggregated data, limiting detailed individual-level analyses<br>▪ Limited sexual behaviour information<br>▪ Do not include illegal abortions (though illegal abortions likely rare in UK)<br>▪ Based on estimates of service users
**Maternity Services Dataset (MSDS)**[36]<br><br>(NHS England)	Secondary, patient-level dataset of information captured through NHS funded maternity services	▪ Comprehensive coverage of NHS maternity services in England<br>▪ Includes all activity carried out in maternity services relating to a mother and baby<br>▪ Includes some demographic information<br>▪ Annual reporting and linkage to some health data sources<br>▪ Comparability of core metrics over time	▪ Limited sexual behaviour information<br>▪ Variable data quality between providers<br>▪ Lack of preconception information<br>▪ Restricted to service users, no data on those not accessing care
**Female Genital Mutilation (FGM) Enhanced Dataset**[37]<br><br>(DHSC)	Mandatory reporting collected from NHS acute trusts, mental health trusts and GP practices in England that includes detailed patient-level information on women and girls identified as having undergone or at risk of FGM	▪ Data collected to support the Department of Health's FGM Prevention Programme<br>▪ Provides detailed clinical and demographic data on FGM prevalence and types<br>▪ Includes some demographic information<br>▪ Quarterly reporting<br>▪ Comparability of core metrics over time	▪ Underreporting likely due to sensitivity, stigma, and variation in identification<br>▪ Potential inconsistencies in coding and recording practices<br>▪ Data completeness and quality vary by reporting provider<br>▪ Restricted to service users, no data on those not accessing care
**Primary and secondary care data sources**			
**Clinical Practice Research Datalink (CPRD)**[38]<br><br>(Medicines & Healthcare Products Regulatory Agency [MHRA])	Secondary dataset of anonymised patient data from a network of general practices (GPs) across the UK, contains longitudinal electronic health records including diagnoses, prescriptions, test results, and referrals	▪ Comprehensive collection of general practice activity and diagnoses, including STI sequalae<br>▪ Includes some demographic, lifestyle information<br>▪ Can link to other administrative data sources (e.g., Hospital Episodes Statistics)<br>▪ Quarterly reporting (dependent on data sharing specification)<br>▪ Includes individual-level, longitudinal data (can build cohort studies)<br>▪ Comparability of core metrics over time (though subject to coverage and coding limitations)	▪ Limited sexual behaviour information<br>▪ Coverage variability, shifts in geographical representation, limited generalisability for smaller geographies<br>▪ Some data quality limitations with introduction or updates to coding (periodic)<br>▪ Limited to reporting of pre-defined activity and diagnosis codes (however, periodically reviewed)<br>▪ Restricted to service users, no data on those not accessing care

Ogaz D, *et al. Sex Transm Infect* 2026; 102:401–408. doi: 10.1136/sextrans-2025-056754

**Hospital Episodes Statistics (HES)**[39]<br><br>(NHS England)	Dataset of patient-level hospital episodes compiled by NHS trusts containing all inpatient, outpatient, and emergency department episodes within NHS hospitals	▪ Comprehensive collection of secondary care (e.g., hospital) activity across multiple care settings<br>▪ Wide service coverage to underpin commissioning<br>▪ Includes some demographic information<br>▪ Can link to other administrative data sources (e.g., CPRD)<br>▪ Quarterly reporting (dependent on data sharing specification)<br>▪ Includes individual-level, longitudinal data (can build cohort studies)<br>▪ Comparability of core metrics over time (though subject to coverage and coding limitations)	▪ Limited sexual behaviour information, lifestyle, socioeconomic factors, and outpatient clinical detail<br>▪ Does not capture care outside NHS hospitals (e.g., private sector), does not include all mental health specific hospital activity<br>▪ Some data quality limitations with introduction or updates to coding (periodic)<br>▪ Limited to reporting of pre-defined activity and diagnosis codes (however, periodically reviewed)<br>▪ Restricted to service users, no data on those not accessing care
**National Emergency Care Data Set (ECDS)**[40]<br><br>(NHS England)	National dataset for urgent and emergency care in England containing detailed patient-level information on all emergency care attendances, including clinical, demographic, and administrative data from emergency departments and urgent care centres	▪ Comprehensive collection of emergency care activity<br>▪ Wide service coverage to underpin commissioning<br>▪ Includes some demographic, lifestyle information<br>▪ Can link to other administrative data sources (e.g., CPRD, HES)<br>▪ Includes episode information<br>▪ Quarterly reporting (dependent on data sharing specification)	▪ Limited sexual behaviour information, lifestyle, socioeconomic factors, and outpatient clinical detail<br>▪ Some data quality limitations with introduction or updates to coding (periodic)<br>▪ Limited to reporting of pre-defined activity and diagnosis codes (however, periodically reviewed)<br>▪ Restricted to service users, no data on those not accessing care
**Census, registries, additional administrative data**			
**Census for England and Wales**[41]<br><br>(ONS)	Population estimates for England and Wales; decennial national survey collecting comprehensive demographic, social, and economic information from all households to provide a detailed population snapshot	▪ Representative of all people and households in England and Wales<br>▪ Decennial reporting, comparability of core estimates over time<br>▪ Enables small-area geographic analyses and population projections<br>▪ Includes some demographic, lifestyle information<br>▪ Consistent methodology, high response rate<br>▪ Comparability of core metrics over time and range of measures to assess inequalities<br>▪ Linkage capability (dependent on data sharing specification)<br>▪ Expanded gender identity questions from 2021	▪ Limited sexual behaviour information<br>▪ Reliance on estimates, imputation<br>▪ Timeliness, processing and release times can delay data availability<br>▪ Further validation of gender identity, sexual orientation metrics required<br>▪ Lack of generalisability to individuals outside of private households
**National Cancer Registry**[42]<br><br>(National Cancer Registration and Analysis Service [NCRAS]/NHS England)	Longitudinal, health-care event-based linked dataset on cancer patients in England, including tumour site, stage, treatment, and outcomes	▪ Comprehensive collection of cancer care pathway activity, includes cancers relevant to sexual health such as cervical, anal, penile, and oropharyngeal cancers<br>▪ Includes some demographic information	▪ Limited sexual behaviour information<br>▪ Some data quality limitations with introduction or updates to coding (periodic)<br>▪ Limited to reporting of pre-defined activity and diagnosis codes (however,

		▪ Linkage capability (dependent on data sharing specification)<br>▪ Annual reporting, comparability of core metrics<br>▪ Includes individual-level, longitudinal data (can build cohort studies)	periodically reviewed), potential inconsistencies in staging or treatment coding across providers<br>▪ Restricted to service users, no data on those not accessing care
**Cervical Screening Statistics**[43]<br><br>(NHS England)	Mandatory dataset of those receiving cervical screening in England, report on coverage, uptake, and outcomes of the NHS Cervical Screening Programme, which aims to detect precancerous changes and prevent cervical cancer	▪ Comprehensive collection of screening activity and outcomes<br>▪ Enables monitoring of programme performance and trends over time, includes some demographic information<br>▪ Linkage capability (dependent on data sharing specification)<br>▪ Annual reporting, comparability of core metrics<br>▪ Includes individual-level, longitudinal data (can build cohort studies)	▪ Limited sexual behaviour information<br>▪ Restricted to service users, no data on those not accessing care<br>▪ Does not capture reasons for non-attendance or access barriers<br>▪ Does not include screening outside the NHS programme (e.g. private providers)
**National Drug and Alcohol Treatment Monitoring System (NDTS)**[44]<br><br>(NHS England)	National data sources containing individual-level data on people receiving publicly funded drug and alcohol treatment, providing information on demographics, treatment episodes, and risk behaviours relevant to sexual health	▪ Comprehensive collection of treatment provider activity that is publicly funded<br>▪ Collects data on risk behaviours including some sexual health-related risks, some demographic information<br>▪ Includes information for key inclusion health population<br>▪ Linkage capability (dependent on data sharing specification)<br>▪ Includes individual-level, longitudinal data (can build cohort studies)	▪ Focused on treatment population; excludes those not in contact with services<br>▪ Limited sexual behaviour information<br>▪ Variable data quality, completeness<br>▪ Restricted to service users, no data on those not accessing care
**Arden and GEM national hepatitis C patient registry and treatment outcome system**[45]<br><br>(NHS England)	Registry commissioned by NHS England to create a national patient register and treatment outcome system for hepatitis C patients	▪ Provides comprehensive, longitudinal clinical data on hepatitis C patients<br>▪ Incentivised data collection, includes data from more than 120 Trusts<br>▪ Includes some demographic information<br>▪ Includes information for key inclusion health population<br>▪ Linkage capability (dependent on data sharing specification)<br>▪ Includes individual-level, longitudinal data (can build cohort studies)	▪ Limited to diagnosed and treated individuals; may miss undiagnosed infections<br>▪ Focused primarily on hepatitis C, limited sexual behaviour information<br>▪ Variable data quality, completeness<br>▪ Coverage variability, shifts in geographical representation<br>▪ Restricted to service users, no data on those not accessing care<br>▪ May lack broader sexual health context outside hepatitis C diagnosis
**Mental Health Services Dataset**[46]<br><br>(NHS England)	Mandatory, patient-level dataset containing detailed, patient-level information on individuals in contact with secondary mental health, learning disability, and autism services	▪ Comprehensive collection of mental health service activity<br>▪ Includes individual-level, longitudinal data (can build cohort studies)<br>▪ Linkage capability (dependent on data sharing specification), can be linked to sexual health data to study intersections between mental and sexual health	▪ Limited sexual behaviour information<br>▪ Data quality varies by provider<br>▪ Some data quality limitations with introduction or updates to coding (periodic)<br>▪ Limited to reporting of pre-defined activity and diagnosis codes (however, periodically reviewed)<br>▪ Restricted to service users, no data on those not accessing care

Ogaz D, *et al. Sex Transm Infect* 2026; 102:401–408. doi: 10.1136/sextrans-2025-056754

**The Adult Psychiatric Morbidity Survey (APMS)**[47]<br><br>(NHS England)	Nationally representative survey of adults in England, conducted periodically, that collects detailed information on mental health conditions, service use, and includes some aspects of sexual behaviour, trauma, and risk	▪ Provides nationally representative data on mental health and associated risk behaviour<br>▪ Includes data on sexual risk behaviours, sexual assault, and related trauma<br>▪ Enables analysis of associations between mental health and sexual health outcomes<br>▪ Linkage capability (dependent on data sharing specification)	▪ Timeliness, conducted infrequently (approximately every 7 years)<br>▪ Sexual health content is limited and not the primary focus
**English Prescribing Dataset (EPD)**[48]<br><br>(NHS England)	Secondary dataset of community issued prescriptions issued and dispensed in England, including contraceptives and treatments for STIs	▪ Comprehensive collection of prescribing and dispensing activity<br>▪ Information based on British National Formulary (BNF) coding<br>▪ Includes some demographic information<br>▪ Linkage capability (dependent on data sharing specification)<br>▪ Supports evaluation of access to contraceptives and STI treatments	▪ Lacks patient-level demographic or clinical detail<br>▪ Limited sexual behaviour information<br>▪ Variable data quality, completeness<br>▪ Some data quality limitations with introduction or updates to coding (periodic)<br>▪ Limited to reporting of pre-defined activity and diagnosis codes (however, periodically reviewed)<br>▪ Restricted to service users, no data on those not accessing care
**Other data sources**			
**English Longitudinal Study of Ageing (ELSA)**[49]<br><br>(UCL)	Longitudinal, cross-sectional population-based survey assessing health, social, wellbeing and economic circumstances in the population aged 50 and older in England, collecting data on health, lifestyle, wellbeing, and socioeconomic circumstances, including limited information on sexual relationships and function	▪ Representative of people aged 50 or over, living in private households in England<br>▪ Provides unique insights into sexual health and relationships in older adults, includes expansive demographic, lifestyle, health behaviour information<br>▪ Sampling based on validated methodology used across national health surveys<br>▪ Comparability of core questions over time<br>▪ Includes some anthropomorphic measurements, biological sampling<br>▪ Includes individual-level, longitudinal data (can build cohort studies)	▪ Limited sexual behaviour information, sexual health data not included in every wave<br>▪ Cross-sectional data collection; sampling, recall, social desirability biases, digital exclusion<br>▪ Low numbers of inclusion health populations<br>▪ Lack of generalisability to individuals outside of private households<br>▪ Cannot establish causality between behavioral data and outcomes of interest
**UK Biobank**[50]<br><br>(UK Biobank Limited)	Biomedical database containing de-identified genetic, lifestyle, health information and biological samples	▪ Comprehensive collection of over 500K participants aged 40-69 at recruitment<br>▪ Large sample size with genetic, biological, and lifestyle data, includes some demographic information<br>▪ Can study biological and social determinants of health outcomes<br>▪ Linkage capability (dependent on data sharing specification)<br>▪ Includes individual-level, longitudinal data (can build cohort studies)	▪ Sexual health data are limited, self-reported and not regularly updated<br>▪ Self-selection bias<br>▪ Underrepresentation of younger adults and marginalised groups<br>▪ Low initial response rate

Ogaz D, *et al. Sex Transm Infect* 2026; 102:401–408. doi: 10.1136/sextrans-2025-056754

		▪ Open access resource	
**National Pupil Database****[51]**<br><br>(Department for Education)<br><br>**note, while not a sexual health data source, this resource captures comprehensive educational information that could inform future evaluation of sexual health education in secondary schools	Individual-level database based on multiple data collections of individuals aged 2-21 in state funded education and higher education in England	▪ Contains detailed individual-level records on educational attainment, attendance, and demographics<br>▪ Includes individual-level, longitudinal data (can build cohort studies)<br>▪ Linkage capability (dependent on data sharing specification), can be linked to health datasets to explore sexual health outcomes in young people	▪ Data linkage requires strict approvals and can be complex to obtain<br>▪ Primarily educational data; limited sexual behaviour information<br>▪ No insights on pupils outside of state school (private schools, home schooled)

Appendix I Reference List

1. The National Survey of Sexual Attitudes and Lifestyles (Natsal) [Available from: https://www.natsal.ac.uk/ accessed 16 December 2024.
2. Johnson AM, Wadsworth J, Wellings K, et al. Sexual lifestyles and HIV risk. *Nature* 1992;360(6403):410–2. doi: 10.1038/360410a0
3. Johnson AM, Mercer CH, Erens B, et al. Sexual behaviour in Britain: partnerships, practices, and HIV risk behaviours. *The Lancet* 2001;358(9296):1835–42. doi: https://doi.org/10.1016/S0140-6736(01)06883-0
4. Sonnenberg P, Clifton S, Beddows S, et al. Prevalence, risk factors, and uptake of interventions for sexually transmitted infections in Britain: findings from the National Surveys of Sexual Attitudes and Lifestyles (Natsal). *Lancet* 2013;382(9907):1795–806. doi: 10.1016/s0140-6736(13)61947-9 [published Online First: 20131126]
5. Dema E, Copas AJ, Clifton S, et al. Methodology of Natsal-COVID Wave 1: a large, quasi-representative survey with qualitative follow-up measuring the impact of COVID-19 on sexual and reproductive health in Britain. *Wellcome Open Res* 2021;6:209. doi: 10.12688/wellcomeopenres.16963.2 [published Online First: 20220328]
6. Dema E, Conolly A, Willis M, et al. Methodology of Natsal-COVID Wave 2: A large, quasi-representative, longitudinal survey measuring the impact of COVID-19 on sexual and reproductive health in Britain. *Wellcome Open Res* 2022;7:166. doi: 10.12688/wellcomeopenres.17850.1 [published Online First: 20240108]
7. Wayal S, Reid D, Blomquist PB, et al. The Acceptability and Feasibility of Implementing a Bio-Behavioral Enhanced Surveillance Tool for Sexually Transmitted Infections in England: Mixed-Methods Study. *JMIR Public Health Surveill* 2018;4(2):e52. doi: 10.2196/publichealth.9010
8. Howarth AR, Saunders J, Reid D, et al. 'Stay at home ...': exploring the impact of the COVID-19 public health response on sexual behaviour and health service use among men who have sex with men: findings from a large online survey in the UK. *Sex Transm Infect* 2022;98(5):346–52. doi: 10.1136/sextrans-2021-055039 [published Online First: 20210920]
9. Aghaizu A, Wayal S, Nardone A, et al. Sexual behaviours, HIV testing, and the proportion of men at risk of transmitting and acquiring HIV in London, UK, 2000-13: a serial cross-sectional study. *Lancet HIV* 2016;3(9):e431–e40. doi: 10.1016/S2352-3018(16)30037-6 [published Online First: 20160714]
10. Logan L, Fakoya I, Howarth A, et al. Combination prevention and HIV: a cross-sectional community survey of gay and bisexual men in London, October to December 2016. *Euro Surveill* 2019;24(25) doi: 10.2807/1560-7917.ES.2019.24.25.1800312 [published Online First: 2019/06/27]
11. Ogaz D, Logan L, Curtis TJ, et al. PrEP use and unmet PrEP-need among men who have sex with men in London prior to the implementation of a national PrEP programme, a cross-sectional study from June to August 2019. *BMC Public Health* 2022;22(1):1105. doi: 10.1186/s12889-022-13425-0 [published Online First: 20220603]
12. The European MSM Internet Survey 2024 [cited 2025 17 January]. Available from: https://www.emis-project.eu/ accessed 17 January 2025.
13. The Reproductive Health Survey for England 2024 [cited 2025 17 January]. Available from: https://www.lshtm.ac.uk/research/centres-projects-groups/reproductive-health-survey-england accessed 17 January 2025.
14. UK Health Security Agency. HIV: positive voices survey 2024 [cited 2025 17 January]. Available from: https://www.gov.uk/government/publications/hiv-positive-voices-survey accessed 17 January 2025.
15. O'Halloran C, Croxford S, Mohammed H, et al. Factors associated with reporting antibiotic use as STI prophylaxis among HIV PrEP users: findings from a cross-sectional online community survey, May-July 2019, UK. *Sex Transm Infect* 2021;97(6):429–33. doi: 10.1136/sextrans-2020-054592 [published Online First: 20201020]
16. NHS England. Health Survey for England 2024 [cited 2025 17 January]. Available from: https://digital.nhs.uk/data-and-information/publications/statistical/health-survey-for-england accessed 17 January 2025.
17. Scottish Government. Scottish Health Survey 2024 [cited 2025 17 January]. Available from: https://www.gov.scot/collections/scottish-health-survey/ accessed 17 January 2025.

Ogaz D, *et al. Sex Transm Infect* 2026; 102:401–408. doi: 10.1136/sextrans-2025-056754

18. Department of Health. Health Survey Northern Ireland 2024 [cited 2025 17 January]. Available from: https://www.health-ni.gov.uk/topics/health-survey-northern-ireland accessed 17 January 2025.
19. Welsh Government. National Survey for Wales 2024 [cited 2025 17 January]. Available from: https://www.gov.wales/national-survey-wales accessed 17 January 2025.
20. Ipsos MORI, NHS England. GP Patient survey 2021 [cited 2023 20 April]. Available from: https://www.gp-patient.co.uk/surveysandreports2021 accessed 20 April 2023.
21. UK Health Security Agency. GUMCAD STI Surveillance System: Data specification and technical guidance 2021 [cited 2023 01 June]. Available from: https://assets.publishing.service.gov.uk/government/uploads/system/uploads/attachment_data/file/1032965/GUMCAD_data_specification_and_technical_guidance_2021.pdf accessed 01 June 2023.
22. Savage EJ, Mohammed H, Leong G, et al. Improving surveillance of sexually transmitted infections using mandatory electronic clinical reporting: the genitourinary medicine clinic activity dataset, England, 2009 to 2013. *Euro Surveill* 2014;19(48):20981. doi: 10.2807/1560-7917.es2014.19.48.20981 [published Online First: 20141204]
23. Mohammed H, Migchelsen SJ, Duffell S, et al. Enhancing surveillance of sexually transmitted infections in England with gender identity and behavioural data: The GUMCAD STI Surveillance System. *Plos One* 2026;21(1):e0341128. doi: 10.1371/journal.pone.0341128
24. UK Health Security Agency. CTAD Chlamydia Surveillance System 2025 [cited 2025 15 January]. Available from: https://www.gov.uk/guidance/ctad-chlamydia-surveillance-system accessed 15 January 2025.
25. UK Health Security Agency. HIV surveillance systems 2008 [cited 2025 15 January]. Available from: https://www.gov.uk/guidance/hiv-surveillance-systems accessed 15 January 2025.
26. UK Health Security Agency. Sentinel Surveillance of Blood Borne Virus (SSBBV) testing 2024 [cited 2025 15 January]. Available from: https://www.gov.uk/government/collections/sentinel-surveillance-of-blood-borne-virus-ssbbv-testing accessed 15 January 2025.
27. UK Health Security Agency. Operational guidance for investigating and managing STI outbreaks 2024 [cited 2025 20 January]. Available from: https://www.gov.uk/government/publications/sexually-transmitted-infections-stis-managing-outbreaks/operational-guidance-for-investigating-and-managing-sti-outbreaks#ukhsa-gastro-data-warehouse.
28. UK Health Security Agency. Gonococcal resistance to antimicrobials surveillance programme report 2024 [Available from: https://www.gov.uk/government/publications/gonococcal-resistance-to-antimicrobials-surveillance-programme-grasp-report accessed 17 December 2024.
29. UK Health Security Agency. Mycoplasma genitalium antimicrobial resistance surveillance (MARS) 2024 [Available from: https://www.gov.uk/government/publications/mycoplasma-genitalium-antimicrobial-resistance-surveillance-mars accessed 17 December 2024.
30. UK Health Security Agency. People who inject drugs: HIV and viral hepatitis monitoring 2024 [cited 2025 15 January]. Available from: https://www.gov.uk/government/publications/people-who-inject-drugs-hiv-and-viral-hepatitis-monitoring accessed 15 January 2025.
31. UK Health Security Agency. UK blood donor survey overview 2024 [2025]. Available from: https://www.gov.uk/government/publications/uk-blood-donor-survey-overview-and-findings.
32. NHS England. Sexual and Reproductive Health Activity Data Set (SRHAD) collection 2024 [cited 2025 15 January]. Available from: https://digital.nhs.uk/data-and-information/data-collections-and-data-sets/data-collections/sexual-and-reproductive-health-activity-data-set-srhad-collection accessed 15 January 2025.
33. NHS England. Infectious diseases in pregnancy screening (IDPS): programme overview 2024 [cited 2025 15 January]. Available from: https://www.gov.uk/guidance/infectious-diseases-in-pregnancy-screening-programme-overview accessed 15 January 2025.
34. Office for National Statistics. Conceptions in England and Wales: 2021 2024 [cited 2025 15 January]. Available from: https://www.ons.gov.uk/peoplepopulationandcommunity/birthsdeathsandmarriages/conceptionandfertilityrates/bulletins/conceptionstatistics/2021 accessed 15 January 2025.

35. Office for Health Improvement and Disparities. Abortion statistics, England and Wales: 2022 2024 [cited 2025 15 January]. Available from: https://www.gov.uk/government/statistics/abortion-statistics-for-england-and-wales-2022 accessed 15 January 2025.
36. NHS England. Maternity Services Data Set (MSDS) 2024 [cited 2025 20 January]. Available from: https://digital.nhs.uk/data-and-information/data-collections-and-data-sets/data-sets/maternity-services-data-set.
37. NHS England. Female Genital Mutilation Datasets 2024 [cited 2025 20 January]. Available from: https://digital.nhs.uk/data-and-information/clinical-audits-and-registries/female-genital-mutilation-datasets accessed 20 January 2025.
38. Medicines and Healthcare Products Regulatory Agency. Clinical Practice Research Datalink 2025 [cited 2025 15 January]. Available from: https://www.cprd.com/ accessed 15 January 2025.
39. NHS England. Hospital Episode Statistics (HES) 2024 [cited 2025 15 January]. Available from: https://digital.nhs.uk/data-and-information/data-tools-and-services/data-services/hospital-episode-statistics accessed 15 January 2025.
40. Abara WE, Bernstein KT, Lewis FMT, et al. Effectiveness of a serogroup B outer membrane vesicle meningococcal vaccine against gonorrhoea: a retrospective observational study. *Lancet Infect Dis* 2022;22(7):1021–29. doi: 10.1016/S1473-3099(21)00812-4 [published Online First: 20220412]
41. Office for National Statistics. Population estimates 2021 [cited 2022 25 September]. Available from: https://www.ons.gov.uk/peoplepopulationandcommunity/populationandmigration/populationestimates accessed 25 September 2022.
42. NHS England. National Cancer Registries and Analysis Service (NCRAS) 2024 [cited 2026 06 March]. Available from: https://digital.nhs.uk/services/data-services-for-commissioners/datasets/national-cancer-registries-and-analysis-service-ncras accessed 06 March 2026.
43. NHS England. Cervical Screening (Annual) 2024 [cited 2025 20 January]. Available from: https://digital.nhs.uk/data-and-information/publications/statistical/cervical-screening-annual.
44. NHS England. National Drug and Alcohol Treatment Monitoring System 2024 [cited 2025 20 January]. Available from: https://digital.nhs.uk/data-and-information/information-standards/information-standards-and-data-collections-including-extractions/publications-and-notifications/standards-and-collections/dapb0107-national-drug-treatment-monitoring-system#:%7E:text=The%20National%20Drug%20and%20Alcohol,providers%20at%20a%20national%20level.
45. NHS England. Developing a clinical registry and outcomes system for hepatitis C 2024 [cited 2025 20 January]. Available from: https://www.ardengemcsu.nhs.uk/showcase/case-studies/case-studies/developing-a-clinical-registry-and-outcomes-system-for-hepatitis-c/.
46. NHS England. Mental Health Services Data Set (MHSDS) 2024 [cited 2025 20 January]. Available from: https://digital.nhs.uk/data-and-information/data-collections-and-data-sets/data-sets/mental-health-services-data-set.
47. NHS England. Adult Psychiatric Morbidity Survey: Survey of Mental Health and Wellbeing, England 2025 [cited 2026 06 March]. Available from: https://digital.nhs.uk/data-and-information/publications/statistical/adult-psychiatric-morbidity-survey accessed 06 March 2026.
48. NHS England. English prescribing data (EPD) 2024 [cited 2025 20 January]. Available from: https://www.nhsbsa.nhs.uk/prescription-data/prescribing-data/english-prescribing-data-epd.
49. English Longitudinal Study of Ageing 2024 [cited 2025 22 January]. Available from: https://www.elsa-project.ac.uk/about-elsa.
50. UK Biobank. UK Biobank 2024 [cited 2025 20 January]. Available from: https://www.ukbiobank.ac.uk/.
51. Department for Education. National Pupil Database 2024 [cited 2025 17 January]. Available from: https://www.find-npd-data.education.gov.uk/categories accessed 17 January 2025.
